# Expression patterns of mismatch repair proteins in cervical cancer uncover independent prognostic value of MSH-2

**DOI:** 10.1136/ijgc-2024-005377

**Published:** 2024-07-01

**Authors:** Madeleine Charlotte van den Berg, Hege F Berg, Tomasz Stokowy, Erling A Hoivik, Kathrine Woie, Hilde Engerud, Akinyemi I Ojesina, Ingfrid Salvesen Haldorsen, Jone Trovik, Bjørn I Bertelsen, Camilla Krakstad, Mari Kyllesø Halle

**Affiliations:** 1 Department of Obstetrics and Gynaecology, Haukeland University Hospital, Bergen, Norway; 2 Centre for Cancer Biomarkers, Department of Clinical Science, University of Bergen, Bergen, Norway; 3 Scientific Computing Group, IT Division, University of Bergen, Bergen, Norway; 4 Department of Obstetrics and Gynecology, Medical College of Wisconsin, Milwaukee, Wisconsin, USA; 5 Medical College of Wisconsin Cancer Center, Medical College of Wisconsin, Milwaukee, Wisconsin, USA; 6 Department of Radiology, Mohn Medical Imaging and Visualization Centre, Haukeland University Hospital, Bergen, Norway; 7 Department of Clinical Medicine, University of Bergen, Bergen, Norway; 8 Department of Pathology, Haukeland University Hospital, Bergen, Norway

**Keywords:** Cervical Cancer, Pathology

## Abstract

**Objective:**

Although early-detected cervical cancer is associated with good survival, the prognosis for late-stage disease is poor and treatment options are sparse. Mismatch repair deficiency (MMR-D) has surfaced as a predictor of prognosis and response to immune checkpoint inhibitor(s) in several cancer types, but its value in cervical cancer remains unclear. This study aimed to define the prevalence of MMR-D in cervical cancer and assess the prognostic value of MMR protein expression.

**Methods:**

Expression of the MMR proteins MLH-1, PMS-2, MSH-2, and MSH-6 was investigated by immunohistochemical staining in a prospectively collected cervical cancer cohort (n=508) with corresponding clinicopathological and follow-up data. Sections were scored as either loss or intact expression to define MMR-D, and by a staining index, based on staining intensity and area, evaluating the prognostic potential. RNA and whole exome sequencing data were available for 72 and 75 of the patients and were used for gene set enrichment and mutational analyses, respectively.

**Results:**

Five (1%) tumors were MMR-deficient, three of which were of neuroendocrine histology. MMR status did not predict survival (HR 1.93, p=0.17). MSH-2 low (n=48) was associated with poor survival (HR 1.94, p=0.02), also when adjusting for tumor stage, tumor type, and patient age (HR 2.06, p=0.013). MSH-2 low tumors had higher tumor mutational burden (p=0.003) and higher frequency of (frameshift) mutations in the double-strand break repair gene *RAD50* (p<0.01).

**Conclusion:**

MMR-D is rare in cervical cancer, yet low MSH-2 expression is an independent predictor of poor survival.

**WHAT IS ALREADY KNOWN ON THIS TOPIC**

- Mismatch repair deficiency (MMR-D) as well as individual MMR protein expression is prognostic in multiple cancer types. Recently, MMR-D has emerged as a marker for immune checkpoint inhibitor response. In cervical cancer the role of MMR proteins remains unclear.

**WHAT THIS STUDY ADDS**

- MMR-D is rare in cervical cancer (1%), although it presents in 30% of neuroendocrine tumors. Moreover, this study reveals low MSH-2 as an independent marker for poor prognosis in cervical cancer. Tumors with low MSH-2 expression associate with higher mutational burden and immune activation as well as p53 abnormalities and *RAD50* frameshift mutations.

**HOW THIS STUDY MIGHT AFFECT RESEARCH, PRACTICE OR POLICY**

- MSH-2 may aid in providing a more complete risk profile within cervical cancer. Additionally, cervical cancer patients with MSH-2 low tumors may be candidates for immune checkpoint inhibitor treatment.

## Introduction

Cervical cancer was the fourth most common cancer in women worldwide in 2022, with 661 021 new cases reported and 348 189 deaths.^1^ Standard treatment is surgery for early-stage disease and concomitant pelvic chemoradiotherapy with brachytherapy in locally advanced disease.^2^ However, 30–40% of patients with advanced disease respond poorly to treatment and few alternative treatment options are available.^3^ Recently, the immune checkpoint inhibitor pembrolizumab was approved by the U.S. Food and Drug Administration (FDA) for use in combination with chemotherapy as first-line treatment in programmed death-ligand 1 (PD-L1)-positive cervical cancers,^4^ and as monotherapy in unselected patients with metastatic and recurrent disease.^5^ Although PD-L1 positivity has shown promise as an immune checkpoint inhibitor response marker,^6^ additional markers are needed to stratify patients more accurately and improve overall response rates.

Patient selection for immune checkpoint inhibitor based on mutational burden or microsatellite instability (MSI) assessment is suggested and often used clinically.^6^ The latter is preferrable due to faster testing time and lower costs. MSI is an accumulation of single-nucleotide mutations and indels due to defects in the DNA mismatch repair (MMR) system, of which four proteins play major roles; MutS homolog 6 (MSH-6), MutS homolog 2 (MSH-2), PMS2 homolog 2 (PMS-2), and MutL homolog 1 (MLH-1). MSH-6 interacts with MSH-2 to form the heterodimer MutSα, whereas PMS-2 dimerizes with MLH-1 to form MutLα. Both complexes are involved in recognizing, excising, and resynthesizing single-base mismatches and indel mispairings. Loss of MMR protein expression (MMR deficiency; MMR-D) leads to high mutational burden and generation of neoantigens, which trigger recruitment of immune cells to the site. These “immune-hot” tumors are more likely to respond to immune checkpoint inhibitor treatment.^6^ MSI is detected clinically either using polymerase chain reaction (PCR) on specific microsatellite markers or by immunohistochemical (IHC) staining of two or four of the MMR proteins MSH-6, MSH-2, PMS-2, and MLH-1. Both methods are easily available, are low cost, and are widely used, facilitating clinical implementation globally.^7^ Response rates to immune checkpoint inhibitors are significantly better in patients with MMR-D tumors, and IHC MMR detection is thus already recommended for selecting patients for immune checkpoint inhibitor treatment for these cancer types.^8^

MMR-D is also suggested as a prognostic marker in colorectal cancer, where MMR-D predicts favorable disease outcome.^9^ In endometrial cancer, no significant difference in survival for MMR-D and MMR-proficient (MMR-P) tumors is reported,^10^ but high levels of MSH-6 independently predict poor survival.^11^ For MSH-2, high expression is related to poor outcome in oral squamous carcinoma.^12^ The prognostic impact of differential MMR expression has, to our knowledge, not previously been explored in cervical cancer. We aimed to determine the prevalence of MMR-D in cervical carcinomas and to explore the predictive power of individual MMR protein expression in a large and prospectively collected cervical cancer cohort.

## Methods

### Patients

A population-based cervical cancer cohort was prospectively collected at Haukeland University Hospital (Bergen, Norway) between 2001 and 2020 as previously described.^13^ Clinical data including age at diagnosis, disease stage, body mass index (BMI), lymph node metastasis, treatment, and follow-up were retrospectively extracted from patient records until March 2021.^14^ Patients were staged according to the International Federation of Gynecology and Obstetrics (FIGO) 2018 guidelines.^15^ Histological type and grade, depth of invasion, inflammation reaction, and vascular space invasion was reviewed by an expert pathologist (BIB) as previously described.^13^

### Immunohistochemistry

The tissue microarrays were constructed from formalin-fixed, paraffin-embedded tissue as previously described^13^ (see online supplemental appendix A and table 1 for staining details).

10.1136/ijgc-2024-005377.supp1 Supplementary data

Scoring of tumor tissue was performed (MCB) blinded to clinicopathological features. To define MMR status, protein expression was scored either as intact or lost nuclear expression. Tumors with loss of one or more MMR proteins were defined as MMR-D. Tumors with lack of internal positive control (positive stromal/immune cells) were excluded from the study. All negative tumors with available tissue (n=7) were further evaluated through staining of full sections to confirm their negative protein status on a larger tumor surface.

To evaluate the prognostic value of individual MMR proteins, sections were also scored using the semi-quantitative staining index method, yielding a score between 0 and 9 (detailed in online supplemental appendix A). In statistical analyses, expression levels were dichotomized based on the best cut-off for prediction of disease-specific survival by the Youden index of the staining index of the specific proteins; cut-off values are shown in online supplemental table 2. To investigate other MMR-D cut-offs described in the cervical cancer literature,^16^ slides were also scored for containing ≤10% or >10% stained cells.

### Transcriptome Analyses

RNA sequencing data for 72 patients was available from our previous study.^17^ All 72 cases were MMR-P. Gene expression analyses of MSH-2 low versus high tumors (n=68) were performed using the J-Express software (Molmine, Bergen, Norway).^18^ High and low expression groups were based on staining index and defined as described in online supplemental table 2. Gene set enrichment analyses were used to identify differentially expressed gene sets between MSH-2 low (SI 0–4) and MSH-2 high (SI 6–9) tumors. Gene set collections of the Molecular Signatures Database v4.0 (MSigDB; Broad Institute, Cambridge, MA, USA), namely Ontology gene sets (C5)^19^ and Hallmark (H)^20^ gene sets, were used to compute overlaps of differentially expressed genes.

### Mutational Analyses

Whole exome sequencing was performed on 75 of the cervical carcinomas. For details regarding DNA extraction, library set up, sequence alignment, and variant calling, see online supplemental appendix A. Total number of sequenced reads, unique reads, covered bases, and coverage per base are summarized in online supplemental table 3. The R/Bioconductor package Maftools (version 2.12.0) was applied to display oncoplots, coBarplot, and lollipopplot2, and the mafCompare and tmb functions were applied to identify differentially mutated genes and mutational burden.^21^ A prespecified list of significantly mutated genes in cervical cancer was applied in the oncoplots.^22^ Mutational burden and gene expression levels were illustrated by boxplots using Statistical Package for the Social Sciences (SPSS) version 26 (SPSS Inc., Chicago, IL, USA).

### Statistical Analysis

Chi-square and Fisher’s exact tests were used to evaluate associations between categorical variables as appropriate. The Mann–Whitney *U* test was used for comparison between groups of continuous variables. Interobserver reliability was analyzed with the (weighted) Cohen’s kappa. Survival was estimated by log-rank (Mantel–Cox) test for group differences and illustrated by Kaplan–Meier curves. Multivariate survival analyses were performed using the Cox’s proportional regression hazard model. Entry date for disease-specific survival analyses was defined as time of primary treatment, and end date was defined as last day of follow-up or death from disease. Statistical significance was defined as p<0.05, and all p-values were two-sided. All statistical analyses were performed in SPSS version 26.

## Results

### Study Population

Sufficient tumor tissue for MMR status assessment was available for 508 patients. Apart from higher FIGO stage, the clinicopathological characteristics of the study cohort represent the full population-based cohort (n=865) (Table 1). Median follow-up time was 62 months. Maximum magnetic resonance imaging (MRI)-assessed tumor diameter was available for 243 patients by reassessment of pelvic MRIs performed as part of the primary workup.^14^

**Table 1:** Clinicopathological characteristics of the study cohorts

Variable	Population-based* (n=865) n (%)	TMAs (n=508) n (%)	P-value†	RNAseq (n=72) n (%)	P-value†	WES (n=75) n (%)	P-value†
Median age (years)			0.410		0.254		0.036
<44	432 (50)	242 (48)		41 (57)		28 (37)	
≥44	433 (50)	266 (52)		31 (43)		47 (63)	
FIGO-18 stage‡			<0.001		<0.001		<0.001
I-IB1	409 (47)	180 (35)		15 (21)		7 (9)	
IB2-IV	456 (53)	328 (64)		57 (79)		68 (91)	
Histological type§			0.194		0.023		0.16
SCC	616 (72)	372 (73)		45 (63)		58 (77)	
AC	199 (23)	101 (20)		18 (25)		11 (15)	
Other	43 (5)	34 (7)		9 (12)		6 (8)	
Grade¶			0.324		0.212		0.55
1/2	590 (84)	430 (85)		56 (78)		63 (86)	
3	116 (16)	72 (14)		16 (22)		10 (14)	
Primary treatment			0.194		<0.001		<0.001
(Chemo)radiation	240 (27)	153 (30)		7 (10)		43 (57)	
Surgery	581 (67)	331 (65)		64 (89)		27 (36)	
Other	44 (5)	24 (5)		1 (1)		5 (7)	

Other histological type: adenosquamous carcinoma, neuroendocrine carcinoma, and undifferentiated tumor. Other primary treatment: palliative treatment without chemotherapy, paclitaxel/carboplatin, 5-fluorouracil/cisplatin, and cisplatin/paclitaxel.

*Prospective cohort collected at Haukeland University Hospital.

†Chi-square in comparison to the population-based cohort.

‡Data are missing from n=4 in the population-based cohort.

§Data are missing from n=7 in the population-based cohort and n=1 in the immunohistochemistry cohort.

¶Data are missing from n=159 in the population-based cohort, n=5 in the immunohistochemistry cohort, n=2 in the RNA sequencing cohort, and n=2 in the WES cohort.

AC, adenocarcinoma; FIGO, International Federation of Gynecology and Obstetrics; IHC, immunohistochemistry; RNAseq, RNA sequencing cohort; SCC, squamous cell carcinoma; TMA, tissue microarray; WES, whole exome sequencing.

**Figure 1:**
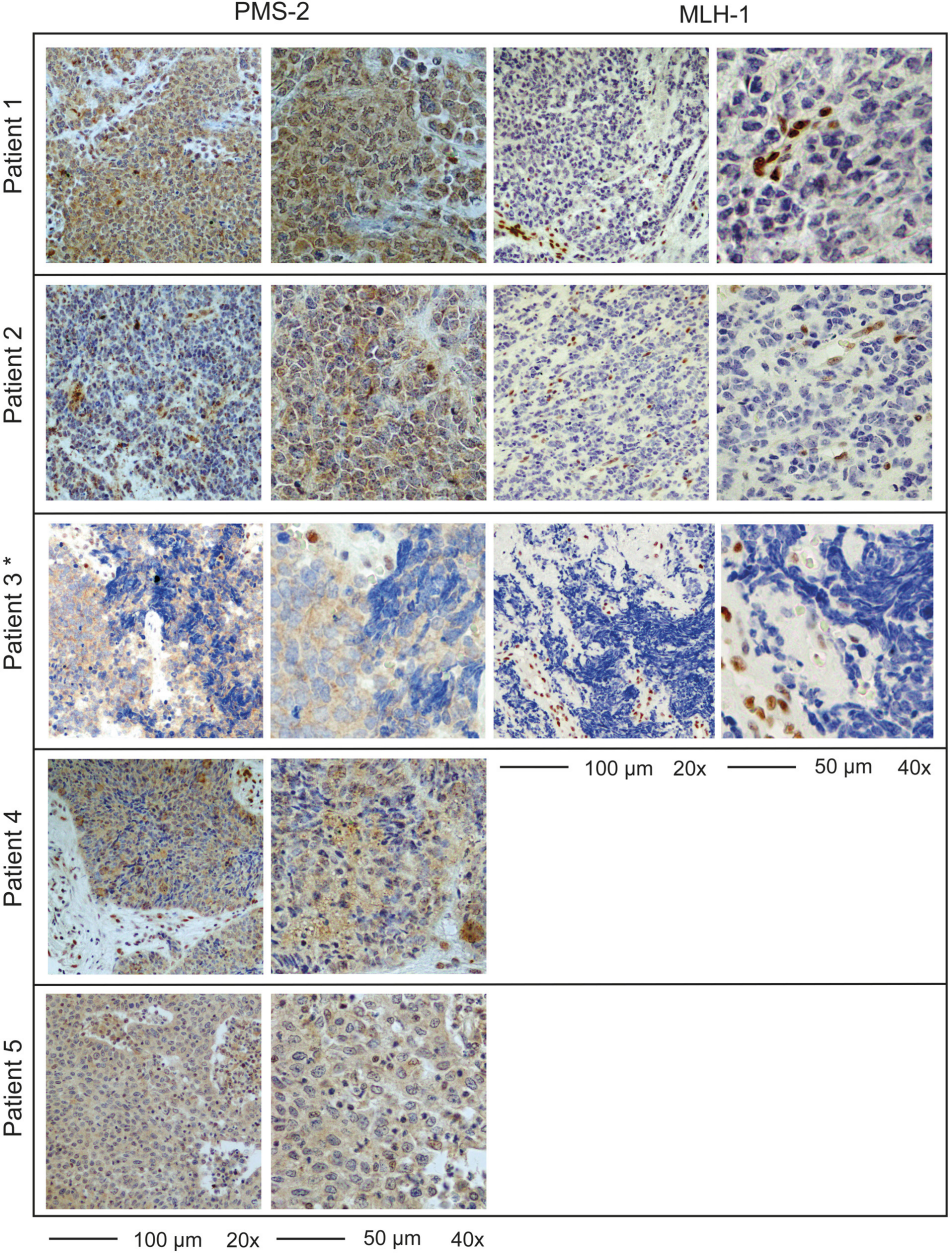
Full section staining of PMS2 and MLH1 validates mismatch repair deficiency (MMR-D) in five patients. Representative images of full sections stained for MMR proteins with negative nuclear staining of tumor cells and positive staining of stroma or immune cells. Although some tumor cells exhibited cytoplasmic staining, no nuclear staining was found. This was confirmed by an expert pathologist. *Tissue block missing, photographs of tissue microarray (TMA).

MMR-D was initially detected in 8 of 508 cervical tumors on tissue microarrays (1.6%). To reduce the risk of falsely classifying the tissue microarray tumor sections as MMR-D, full sections of these tumors were re-stained resulting in three tumors being reclassified as MMR-P. Within the remaining five MMR-D tumors, all had loss of PMS-2, and three had combined loss of MLH-1 and PMS-2 (Figure 1, online supplemental table 4). No significant association between MMR-D and disease-specific survival was found (p=0.17; online supplemental figure 1A). Rescoring using <10% as cut-off for MMR-D was also not prognostic (online supplemental figure 1B). The prevalence of MMR-D was highest within the rare and very aggressive neuroendocrine carcinomas. Three of ten (30%) neuroendocrine carcinomas were MMR-D, representing 60% of all MMR-D cases (online supplemental table 5). Proficient and deficient neuroendocrine carcinomas had similar 5-year disease-specific survival (online supplemental figure 2).

**Figure 2:**
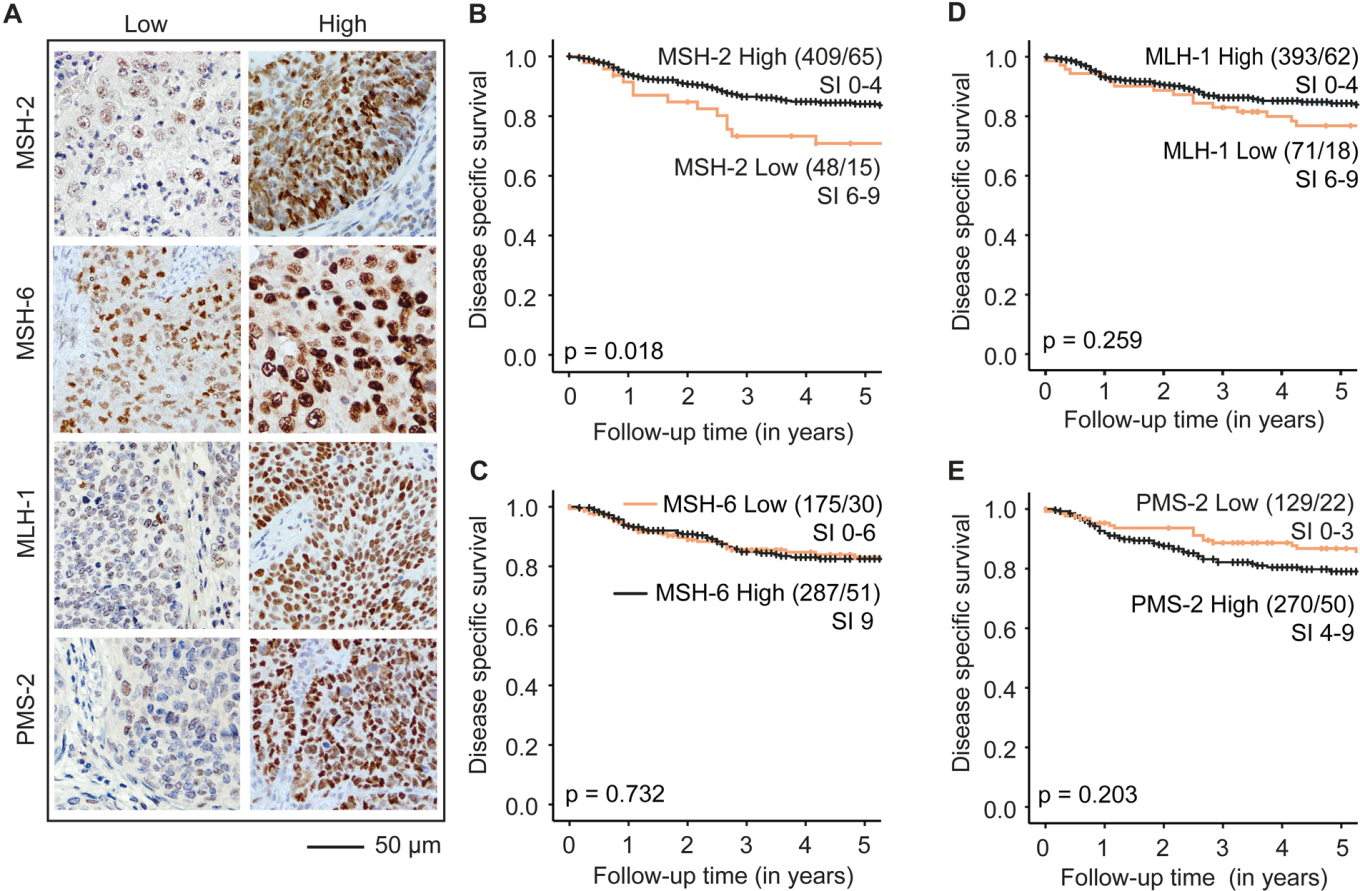
Differential expression of MSH-2, but not MSH-6, MLH-1, and PMS-2, associates with disease-specific survival in cervical cancer. (A): Tumors were defined as ‘low’ or ‘high’ for each of the mismatch repair (MMR) proteins. Low and high MMR protein expression was defined based on staining index (SI) 0–9. (B–E) Disease-specific survival relative to MSH-2 (B), MSH-6 (C), MLH-1 (D), and PMS-2 (E) protein levels. Best cut-off for predicting disease-specific survival (Youden index) was applied (MSH-2: ‘low’ SI 0–4 and ‘high’ SI 6–9, MLH-1: ‘low’ SI 0–6 and ‘high’ SI 9, MSH-6: ‘low’ SI 0–6 and ‘high’ SI 9, PMS-2: ‘low’ SI 0–3 and ‘high’ SI 4–9). P-values are given by log-rank (Mantel–Cox) test. Numbers in parentheses indicate total number of patients/events.

To evaluate the prognostic value of individual MMR proteins, differential expression of MSH-6, MSH-2, PMS-2, and MLH-1 was scored from tissue microarrays (n=508) using the staining index method (Figure 2A). Weighted kappa scores were calculated from independently scoring (MCB and MKH) of a subset of cases demonstrating overall good concordance: k=0.826 for MLH-1 (n=65), k=0.839 for MSH-2 (n=63), k=0.771 for MSH-6 (n=66), and k=0.703 for PMS-2 (n=60).

High and low expression were defined from the Youden index as described in the Methods section. MSH-2 low (staining index 0–4) associated with poor disease-specific survival (p=0.018) (Figure 2B), whereas differential expression of MSH-6, MLH-1, or PMS-2 did not associate with survival (all p>0.05) (Figure 2C–E). MSH-2 protein level did not associate with any clinicopathological variables except for p53 status (online supplemental table 6). However, MSH-2 low independently predicted poor disease-specific survival after adjusting for age, FIGO stage, and histological type (HR 1.77, 95% CI 1.00 to 3.17, p=0.049) (Table 2). To account for possible interactions between age, FIGO stage, and histological type, interaction terms age*FIGO stage, FIGO stage*histological type, and histological type*age were explored. None influenced the effect. To evaluate the reproducibility and applicability of low MSH-2 tumor expression as a potential biomarker in future clinical settings, interobserver agreement for scoring MSH-2 low versus MSH-2 high was analyzed. Kappa agreement for categorizing MSH-2 in high or low was almost perfect (k=0.924).

**Table 2:** Multivariate survival analysis of patients (n=457*) with high MSH-2 versus low MSH-2 staining index according to Cox’s proportional hazard regression method.

Variable	Unadjusted			Adjusted		
	HR	95% CI	P-value	HR	95% CI	P-value
MSH-2 ≤4	1.94	1.11 to 3.41	0.020	1.77	1.00 to 3.13	0.049
Age	1.04	1.03 to 1.05	<0.001	1.03	1.02 to 1.05	<0.001
FIGO-18 ≥IB2	7.60	4.60 to 12.57	<0.001	12.82	4.03 to 40.80	<0.001
Histological type						
Squamous cell carcinoma						<0.001
Adenocarcinoma	0.71	0.38 to 1.33	0.286	1.41	0.70 to 2.85	0.34
Other	3.55	2.07 to 6.100	<0.001	3.71	2.08 to 6.59	<0.001

Other histological type: adenosquamous carcinoma, neuroendocrine carcinoma, and undifferentiated carcinoma.

*Only cases with available data for all variables in the multivariate analyses were included in the univariate analyses.

CI, confidence interval; FIGO, International Federation of Gynecology and Obstetrics; HR, hazard ratio.

**Figure 3:**
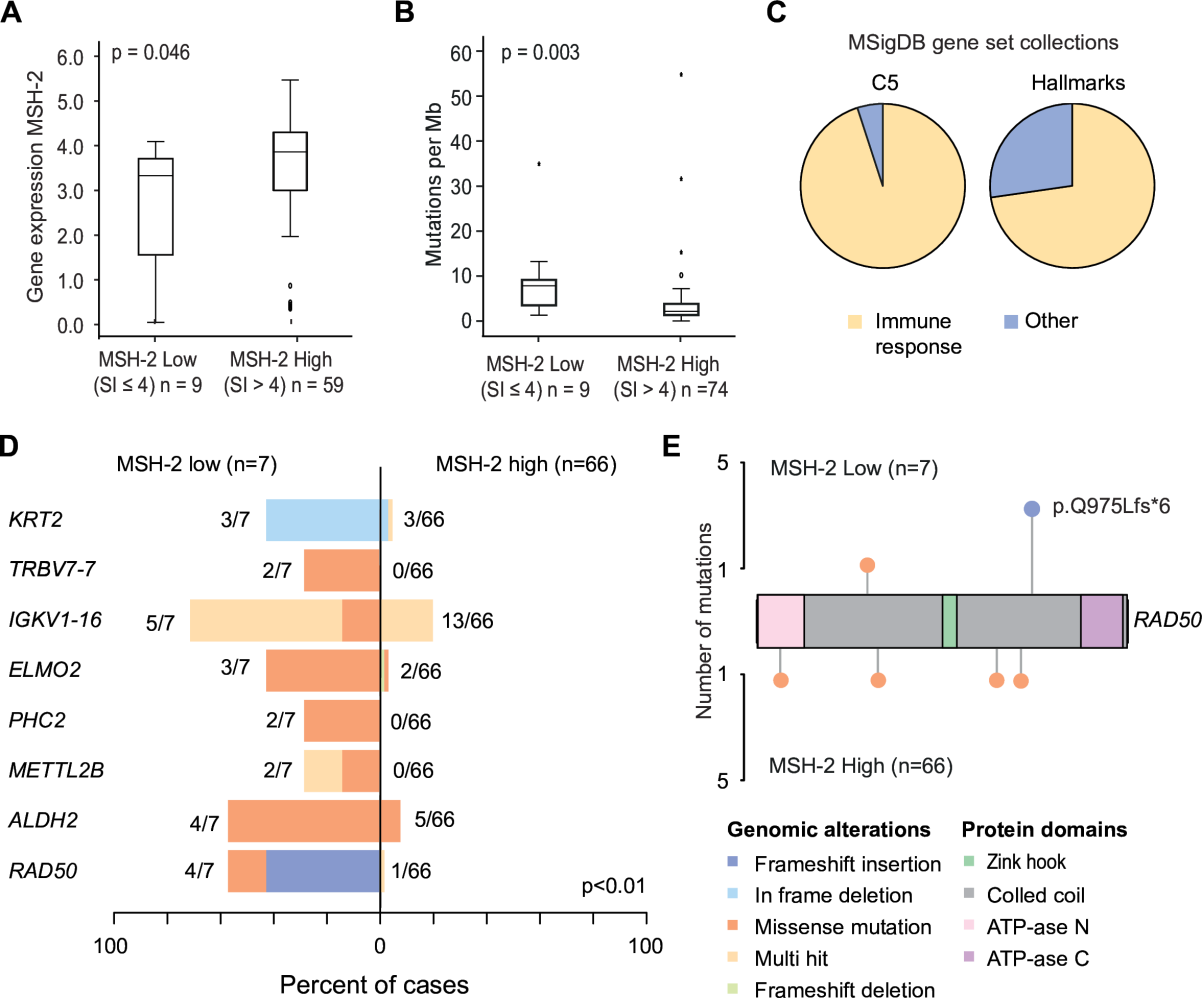
Transcriptomic and genomic characterization reveal high mutational burden and immune cell signaling in MSH-2 low tumors. (A/B) Immunohistochemical protein expression (MSH-2: SI 0–4 and SI 6–9) in relation to corresponding MSH-2 mRNA expression (A) and mutational load (B). (C) Gene set enrichment analysis showing distribution of top 20 ranked enriched gene sets within the C5 and Hallmark gene set collections (MSigDB) for tumors with low MSH-2 expression. Other Hallmark gene sets: ‘coagulation’, ‘KRAS signaling’, ‘p53 pathway’. Other C5 gene sets: ‘cornification’. (D) Top eight differentially mutated genes of MSH-2 low tumors compared with MSH-2 high tumors. Multiple mutations per case per gene is indicated as ‘multi-hit’. Numbers indicate frequencies of patients with mutations and percentages are indicated on the bar below. (E) *RAD50* co-lollipop plot illustrating type and location of mutations. Three of four *RAD50* mutations in MSH-2 low tumors were frameshift mutations at location p.Q975Lfs*6. In MSH-2 high all four missense mutations were detected within the same patient tumor. P-values are given by Mann–Whitney *U* test. SI, staining index.

RNA sequencing and whole exome sequencing data were available for 72 and 75 patients, respectively. *MSH-2* mRNA levels were significantly correlated with MSH-2 protein expression in overlapping samples (p=0.046, n=68) (Figure 3A). MSH-2 low tumors (n=9) had a higher mutational burden compared with MSH-2 high tumors (p=0.003, n=74) (Figure 3B). In gene set enrichment analyses, gene sets related to immune activation were enriched in MSH-2 low tumors. Among the 20 top-ranked gene sets in MSH-2 low tumors, 90% in the Ontology gene sets (C5) and 70% of Hallmark gene sets were related to immune response (GO ‘adaptive immune response’, ‘T-cell activation’ and Hallmark ‘interferon gamma response’, ‘inflammatory response’ (Figure 3C, online supplemental table 7). In MSH-2 high tumors, none of the enriched gene sets were related to immune response.

Mutational analyses grouped for MSH-2 low (n=7) versus MSH-2 high (n=66) revealed a significantly higher mutational frequency in MSH-2 low tumors (p<0.001) (Figure 3C). Eight genes (*KRT2, TRBV7-7, IGKV1-16, ELMO2, PHC2, METTL2B, ALDH2,* and *RAD50*) had significantly higher mutation frequency in MSH-2 low tumors (p>0.01) (Figure 3D). *RAD50* mutations were previously correlated to survival in other cancer types^23 24^ and were therefore further explored, revealing a recurrent (n=4) frameshift insertion mutation in MSH-2 low tumors (Figure 3E).

## Discussion

### Summary of Main Results

Loss of MMR proteins is a common event in cancer and has been proposed as a marker for response to immune checkpoint inhibitors for several cancer types.^8^ In cervical cancer, the frequency and role of MMR loss is not clearly determined. We herein demonstrate that MMR loss is extremely rare in cervical cancer. Only 1% (n=5) of patients showed complete loss of one or more MMR proteins. When examining levels of MMR proteins individually, we found that MSH-2 low independently predicted poor outcome in cervical cancer and that MSH-2 low associated with higher mutational burden, *RAD50* frameshift mutations, and an immune reactive transcriptome.

### Results in the Context of Published Literature

Three of the five (60%) MMR-deficient tumors were of neuroendocrine histology which accounts for 33% (3/10) of all cervical neuroendocrine carcinomas included in this study. Other studies have found similar levels of MMR-D in cervical neuroendocrine carcinomas (30%, n=20^25^ and 33%, n=9^26^). Neuroendocrine carcinomas are aggressive tumors that are challenging to characterize due to their rareness and, to date, no effective treatment regimens exist. As MMR-D is a successful biomarker for immune checkpoint inhibitor response,^8^ the present and previous studies could indicate that immune checkpoint inhibitors could be a valid treatment option for up to a third of these neuroendocrine carcinoma patients.

Regarding MMR-D levels, our findings contrast with previous studies where substantially higher MMR-D frequencies were found, generally ranging from 11% to 22% (n=102–186) of cervical cancers.^27–29^ This may be partly due to different sample size as well as the different detection and scoring methods used. Noh et al^27^ used both IHC and PCR to identify MMR-D (observing 11.3% MMR-D and/or MSI, without further specification) and Nijhuis et al^29^ used full sections with a lower cut-off (1%) for MMR loss (observing 22% MMR-D). However, none of these studies reported positive stroma cells as an internal control for MMR expression. Consistent with our findings, Bonneville et al analyzed whole exome sequencing data from The Cancer Genome Atlas (TCGA) cervical cancer cohort (n=305) and identified MSI in 2.6% of the tumors.^30^ We did not detect any significant differences in survival between patients with MMR-deficient and MMR-proficient tumors, which is in line with previous reports.^27 30^

This study reveals low MSH-2 as an independent marker for poor prognosis in cervical cancer. Furthermore, our study reveals that 80% of the MSH-2 low tumors had aberrant p53 staining. We have previously shown that aberrant p53 associates with poor survival.^13^ p53 is a major apoptotic regulator in cancer.^31^ MMR proteins, and specifically MSH-2, have also been found to influence apoptotic signaling by recognizing damaged DNA independently of their repair function.^32^ Thus, a combination of impaired MSH-2 and aberrant p53 may lead to increased mutagenesis and disrupted apoptotic signaling. We did indeed identify a significantly higher mutational burden in MSH-2 low versus high tumors, suggesting that a low level of MSH-2 may impair DNA MMR thereby inducing a higher mutational load, regardless of expression levels of the other MMR proteins. This is supported by a previous study demonstrating that a reduction of *MSH-2* mRNA expression by 25% or more significantly decreases MMR efficacies,^33^ yet for *MLH-1* a 50% decrease is necessary to find similar effects.^33^

A recurrent *RAD50* p.Q975Lfs*6 frameshift insertion was detected in four of seven MSH-2 low tumors with available mutational data. Pan-cancer analysis has revealed that *RAD50* is the most frequently altered gene in MSI tumors.^34^ Also, mutations in and/or low expression of RAD50 have been associated with poor survival in multiple cancer types.^23 24^ RAD50 is part of the DNA damage repair Mre11-Rad50-Nbs1 (MRN)-complex and depletion links to double-strand break accumulation and increased apoptosis independent of p53.^35^ Loss of RAD50 function may thus play a role in the tumorigenesis of these tumors.

### Strengths and Weaknesses

In our study all negative TMA tumor specimens were re-stained using full sections and verified by a trained pathologist to ensure true negative lesions. This is to date the largest cervical cancer study of MMR-D protein levels and their relationship to patient outcomes. Still, despite the comprehensive and population-based nature of this study, the genomic and clinicopathological analyses were hampered by a limited number of MMR-D and MSH-2 low patients.

### Implications for Practice and Future Research

To our knowledge, low MSH-2 has not previously been identified as an independent prognostic marker for poor survival. Whether MSH-2 could aid in providing a more complete risk profile within cervical cancer subgroups warrants further investigation. Mutational burden is one hallmark of immune checkpoint inhibitor response alongside immune activation,^6^ and in our genomic analyses we found higher mutational burden and enriched immune signaling in the MSH-2 low tumors. If our findings are confirmed in follow-up studies, cervical cancer patients with MSH-2 low tumors may be candidates for immune checkpoint inhibitor treatment. Furthermore, this is the first discovery of the *RAD50* p.Q975Lfs*6 frameshift mutation in cervical cancer. A possible link between RAD50 and the MMR system should be further investigated in cervical cancer.

## Conclusions

This comprehensive characterization of MMR protein expression confirms that MMR-D is rare in cervical cancer, yet in neuroendocrine tumors MMR-D is abundant. Low expression of the MMR protein MSH-2 is discovered as an independent poor prognosis marker and associates with high mutational burden, immune activation, and *RAD50* frameshift mutations. Together these findings indicate that MSH-2 low and not MMR-D could be considered as an immune checkpoint inhibitor response marker in cervical cancer. As our results are impaired by a limited number of MSH-2 low tumors (n=48), the clinical applicability of MSH-2 as a prognostic and predictive marker warrants further validation in multi-institutional studies.

## Data Availability

Data are available upon reasonable request. In accordance with the journal’s guidelines, we will provide our data for independent analysis by a selected team by the Editorial Team for the purposes of additional data analysis or for the reproducibility of this study in other centers if such is requested.
