# Supplementary material for: Expression patterns of mismatch repair proteins in cervical cancer uncover independent prognostic value of MSH-2
Source: Int J Gynecol Cancer. 2024 Jul 1;34(7):993–1000. doi: 10.1136/ijgc-2024-005377 (PMC11228214; doi:10.1136/ijgc-2024-005377)
Supplement: Supplementary data [file ijgc-2024-005377supp001.pdf]

BMJ Publishing Group Limited (BMJ) disclaims all liability and responsibility arising from any reliance placed on this supplemental material which has been supplied by the author(s)

Expression patterns of MMR proteins in cervical cancer uncover independent prognostic value of MSH-2

Supplementary material

Madeleine C. van den Berg[1,2], MD, Hege F. Berg[1,2], PhD, Tomasz Stokowy[3], PhD, Erling A. Hoivik[1,2,], PhD, Kathrine Woie[1], MD, PhD, Hilde Engerud[1,2], MD, PhD, Akinyemi I Ojesina[4,5], MD, PhD, Ingfrid S. Haldorsen[6,7], MD, PhD, Jone Trovik[1,2], MD, PhD, Bjørn I. Bertelsen[8], MD, PhD, Camilla Krakstad[1,2], PhD and Mari Kyllesø Halle[1,2, #], PhD

[1]Department of Obstetrics and Gynaecology, Haukeland University Hospital, Bergen, Norway;
[2]Centre for Cancer Biomarkers, Department of Clinical Science, University of Bergen, 5053 Bergen, Norway.
[3] Scientific Computing Group, IT Division, University of Bergen Norway.
[4] Department of Obstetrics and Gynecology, Medical College of Wisconsin, Milwaukee, WI, USA.
[5]Medical College of Wisconsin Cancer Center, Medical College of Wisconsin, Milwaukee, WI, USA.
[6]Department of Clinical Medicine, University of Bergen, Bergen, Norway.
[7]Mohn Medical Imaging and Visualization Centre, Department of Radiology, Haukeland University Hospital, Bergen, Norway.
[8]Department of Pathology, Haukeland University Hospital, 5021 Bergen, Norway
[#]Corresponding author:
Mari Kyllesø Halle: Glasblokkene, Blokk 1, Haukelandsbakken 15, 5009 Bergen; e-mail: mari.halle@uib.no; phone: +47 99 59 65 97; ORCID: 0000-0002-2660-8271

Appendix A

Methods

Patient cohort

MRIs in this study include T2-weighted sequences acquired and read in two orthogonal planes.

Immunohistochemical staining

TMA slides were dewaxed in xylene, rehydrated in graded ethanol prior to antigen retrieval in pH 9 (S-2367; Dako Denmark A/S, Glostrup, Denmark) or pH 6 (S-1699 Dako, North America) by boiling for 15 minutes. Endogenous peroxidase activity was blocked (S-2023; Dako Denmark A/S) and sections were stained for 30 min (anti MLH-1, anti MSH-2) or one hour (anti MSH-6, anti PMS-2) at room temperature with primary antibodies

Sections were then stained with horseradish peroxidase-labeled secondary anti-mouse antibody (K-4001; Dako-Denmark A/S) for 30 minutes. Finally, DAB-chromogen (K-4010; Dako Denmark A/S) was applied, the sections were counterstained with hematoxylin and dehydrated in ethanol and xylene before mounting.

Evaluation of scoring

Sections were scored using the semi-quantative staining index (SI) method, considering both staining intensity and area of positive staining. Intensity of nuclear staining was scored as 0 = negative, 1 = weak, 2 = moderate or 3 = strong, and area of the staining was scored 0 = 0%, 1 = 1-10%, 2 = 10-50% and 3 > 50% positive staining. The SI was calculated as a product of intensity and area.

Transcriptomic analyses

GSEAs were performed within the JExpress software applying Golub (signal to noise) as scoring method and by permuting on genes (1000 permutations).

Mutational analyses

DNA was extracted from snap-frozen tumor tissue by using the AllPrep DNA/RNA Mini kit (Qiagen, Germany) in agreement with the manufacturer's protocol. The libraries were set up using KAPA Hyper Prep (100 ng input) and captured using the SeqCap EZ MedExome

(Roche, Basel, Switzerland). The Illumina HiSeq 4000 (Twist: 100 × 2) was applied for sequencing. Bwa-mem (v.0.7.17) was used to align sequences to the human genome GRCh38, executed using the dockstore-cgpmap 3.3.0 pipeline. Raw reads quality was assessed using the FASTQC and Picard software (v.2.17.0). The GATK was applied for depth and coverage analyses. MultiQC (was used to generate data quality report and control factors such as duplicate and alignment rate. Total number of sequenced reads, unique reads, covered bases and coverage per base are summarized in Supplementary Table 3). Variant calling procedure included consideration of a 1000 genomes panel of normal samples (suggested by Mutect documentation), calculation of contamination tables and finally filtering using FilterMutectCalls function. From the filtered calls we extracted variants which had “PASS” annotation. Overlap of called variants was limited to exome regions using MedExome hg38 bed files from Roche and ontarged capture with bedtools intersect (version 2.27.1). Variants were annotated using Ensembl VEP following vcf2maf instruction 1.6.21.

Supplementary figures

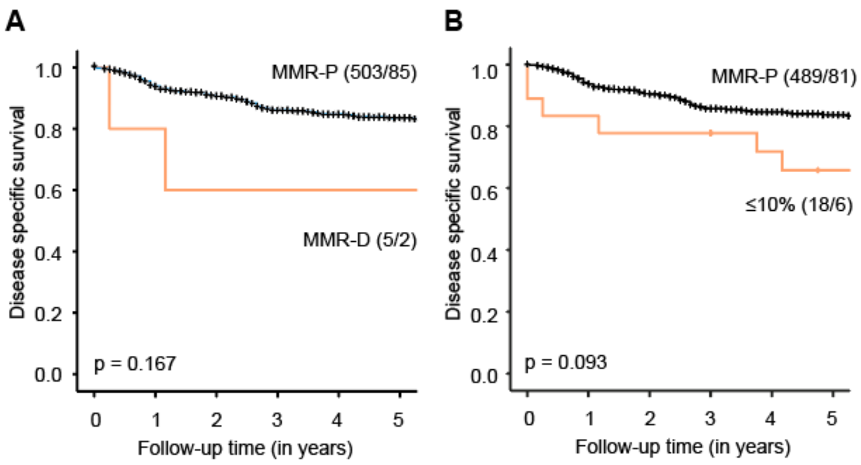

Supplementary figure 1 - No difference in survival between patients with MMR-deficient versus proficient tumors

A/B: Mismatch repair deficiency (MMR-D) did not associate with disease specific survival (A) or ≤ 10% MMR positive cells (B). P-values are given by log-rank (Mantel-Cox) test. Number in brackets indicates total number of patients/events.

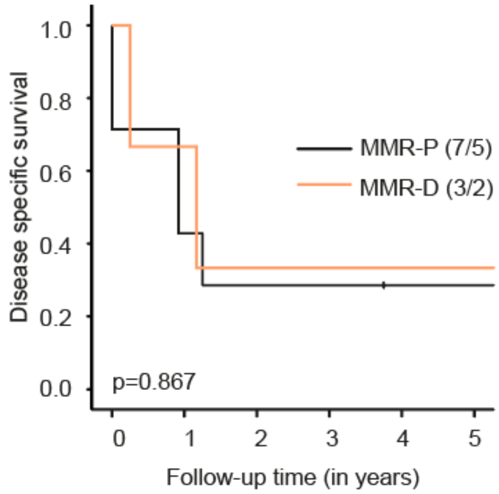

Supplementary figure 2 - MMR-deficiency is not prognostic within patients with neuroendocrine carcinomas

No difference in survival outcomes were detected between patients with mismatch repair deficient (MMR-D) and mismatch repair proficient (MMR-P) neuroendocrine carcinomas. P-values are given by log-rank (Mantel-Cox) test. Number in brackets indicates total number of patients/number of events.

Supplementary tables

Supplementary table 1– Immunohistochemistry antibody information.

Primary antibody	Supplier	Catalogue number	Dilution	Incubation time	HIER buffer
**MLH-1**	Leica Biosystems	MLH1-L-CE	1 : 100	30 min	pH 9
**MSH-6**	Leica Biosystems	MSH6-L-CE	1 : 25	60 min	pH 9
**MSH-2**	Leica Biosystems	MSH2-L-CE	1 : 50	30 min	pH 6
**PMS-1**	Leica Biosystems	PMS2-L-CE	1 : 30	60 min	pH 9

HIER: Heat induced epitope retrieval

Supplementary table 2 – Cut-off values for the dichotomization of the staining index for the MMR proteins

Protein	Low<br>Range staining index (n)	High<br>Range staining index (n)
MLH-1	0-6 (232)	9 (232)
MSH-2	0-4 (48)	6-9 (409)
MSH-6	0-6 (175)	9 (287)
PMS-2	0-3 (177)	4-9 (272)

Supplementary table 3 – sequencing coverage and quality statistics of the whole exome sequencing

Sample ID	Total number of sequenced reads	Total number of uniquely mapped non-duplicate reads[a]	Total number of covered bases (Mb)[b]	Median coverage per base[b]	Minimum coverage per base[b]	Maximum coverage per base[b]	Percentage of targeted bases with coverage ≥30[b,c,d]
T_001	169600000	110017870	11040.4	117.0	0	4773	99.0%
T_002	168000000	110679618	11103.0	115.0	0	2632	99.0%
T_003	183000000	118643264	11890.7	125.0	0	2694	99.0%
T_004	200000000	127458930	12797.0	134.0	0	3175	99.0%
T_005	165000000	108306450	10873.3	116.0	0	2615	99.0%
T_006	191600000	123306116	12359.3	129.0	0	2383	99.0%
T_007	199200000	126689030	12702.1	134.0	0	2955	99.0%
T_008	196400000	123106042	12324.4	129.0	0	3112	99.0%
T_009	203800000	130482752	13081.0	138.0	0	2446	99.0%
T_010	174400000	113401606	11380.7	121.0	0	2683	99.0%
T_011	198800000	127301438	12751.2	137.0	0	2866	99.0%
T_012	203400000	129176532	12962.0	140.0	0	2988	99.0%
T_013	216600000	136701680	13711.4	147.0	0	3085	99.0%
T_014	181800000	120305458	12073.3	129.0	0	2836	99.0%
T_015	163200000	107128796	10745.5	115.0	0	2800	99.0%
T_016	178200000	113999368	11438.6	122.0	0	2738	99.0%
T_017	183600000	124211754	12436.7	127.0	0	3595	99.0%
T_018	203400000	134600872	13456.6	142.0	0	2822	99.0%
T_019	172600000	116187898	11644.5	122.0	0	2664	99.0%
T_020	182200000	120234622	12049.2	127.0	0	2352	99.0%
T_021	210200000	136447406	13684.9	139.0	0	3025	99.0%
T_022	164600000	111760200	11198.7	117.0	0	2985	99.0%
T_023	188600000	126147470	12655.4	133.0	0	3324	99.0%
T_024	171800000	117133020	11746.8	123.0	0	2403	99.0%
T_025	196600000	134752866	13495.8	143.0	0	2965	99.0%
T_026	241200000	154958410	15526.1	161.0	0	3509	99.0%
T_027	179600000	124554334	12481.0	128.0	0	2505	99.0%
T_028	187400000	127697606	12810.2	133.0	0	2879	99.0%
T_029	200400000	138662390	13922.2	147.0	0	3358	99.0%
T_030	196000000	134378194	13474.7	132.0	0	3769	99.0%
T_031	198200000	135821092	13608.9	138.0	0	3225	99.0%
T_032	194200000	134602558	13485.1	140.0	0	3165	99.0%
T_033	228600000	144664250	14520.3	152.0	0	3923	99.0%
T_034	233200000	148220172	14874.9	158.0	0	3388	99.0%
T_035	212000000	135444592	13603.7	143.0	0	3198	99.0%
T_036	216600000	139919874	14032.1	148.0	0	3288	99.0%
T_037	209600000	134680836	13525.4	143.0	0	3408	99.0%

T_038	195400000	127902098	12840.5	131.0	0	2555	99.0%
T_039	236200000	144312316	14488.3	152.0	0	3519	99.0%
T_040	197000000	127271692	12776.4	136.0	0	2899	99.0%
T_041	214200000	138653710	13936.1	142.0	0	3444	99.0%
T_042	229800000	149497846	15024.0	152.0	0	4242	99.0%
T_043	206800000	135137030	13574.8	136.0	0	3815	99.0%
T_044	188600000	125706200	12638.4	124.0	0	2863	99.0%
T_045	198600000	133914954	13457.3	134.0	0	3240	99.0%
T_046	198200000	131075234	13173.2	136.0	0	3366	99.0%
T_047	200000000	137953264	13864.3	139.0	0	3841	99.0%
T_048	220200000	140855838	14144.6	143.0	0	3548	99.0%
T_049	215400000	143900282	14450.3	137.0	0	3741	99.0%
T_050	232400000	151221634	15191.4	144.0	0	3755	99.0%
T_051	194200000	132790508	13338.7	125.0	0	3259	99.0%
T_052	229600000	151089920	15167.8	141.0	0	3990	99.0%
T_053	230200000	154875352	15564.5	149.0	0	4106	99.0%
T_054	230200000	147943904	14858.0	132.0	0	5514	99.0%
T_055	186400000	128148624	12861.6	121.0	0	3646	99.0%
T_056	228000000	148965838	14951.6	141.0	0	3479	99.0%
T_057	222400000	150455170	15115.3	145.0	0	3261	99.0%
T_058	192800000	134842330	13560.2	130.0	0	3559	99.0%
T_059	224000000	151819424	15260.4	143.0	0	3976	99.0%
T_060	220400000	145850496	14657.0	138.0	0	4061	99.0%
T_061	203800000	138659540	13941.9	131.0	0	3478	99.0%
T_062	198600000	138587462	13906.4	132.0	0	3331	99.0%
T_063	201600000	137285790	13797.0	133.0	0	3833	99.0%
T_064	208200000	143024052	14370.2	137.0	0	3370	99.0%
T_065	210200000	133203104	13385.8	130.0	0	3203	99.0%
T_066	222400000	141011524	14168.1	135.0	0	3911	99.0%
T_067	202600000	129025618	12969.2	128.0	0	3157	99.0%
T_068	229800000	146906172	14766.2	147.0	0	3427	99.0%
T_069	210000000	136790902	13747.1	134.0	0	3452	99.0%
T_070	212600000	137222510	13775.4	136.0	0	2822	99.0%
T_071	217200000	138697614	13945.6	134.0	0	2964	99.0%
T_072	204400000	131754672	13224.7	128.0	0	3495	99.0%
T_073	205000000	135307520	13588.9	134.0	0	3681	99.0%
T_074	189400000	123665818	12422.8	122.0	0	3169	99.0%
T_075	201000000	134447212	13494.1	133.0	0	3680	99.0%
T_076	172400000	116111036	11659.5	114.0	0	2769	99.0%
T_077	182800000	122638820	12315.4	121.0	0	2852	99.0%
T_078	179200000	119182970	11960.0	117.0	0	2719	99.0%
T_079	169400000	113793812	11432.6	110.0	0	2820	99.0%
T_080	192600000	126132452	12660.6	123.0	0	2782	99.0%

T_081	170800000	120428180	12066.7	121.0	0	3536	99.0%
T_082	180400000	126019104	12590.9	129.0	0	3114	99.0%
T_083	162200000	114276034	11449.0	114.0	0	3801	99.0%
T_084	176400000	121996642	12221.3	123.0	0	2470	99.0%
T_085	168800000	118489876	11862.2	117.0	0	3218	99.0%
T_086	199800000	135633000	13579.2	136.0	0	3576	99.0%
T_087	253600000	158775516	15905.1	156.0	0	6627	99.0%
T_088	159800000	112448938	11268.7	112.0	0	2989	99.0%
T_089	218800000	143000914	14324.1	143.0	0	3533	99.0%
T_090	205000000	134447504	13474.2	133.0	0	3328	99.0%
T_091	196400000	133809434	13402.5	130.0	0	3612	99.0%
T_092	188200000	128890670	12887.7	125.0	0	3136	99.0%
T_093	164000000	115805900	11591.9	115.0	0	2807	99.0%
T_094	190200000	129754692	12978.2	128.0	0	4321	99.0%
T_095	176400000	121905918	12204.1	119.0	0	3607	99.0%
T_096	196800000	132049776	13215.2	131.0	0	3259	99.0%
N_001	110200000	76690606	7688.0	82.0	0	2077	99.0%
N_002	98400000	70530462	7067.0	76.0	0	1860	98.0%
N_003	96600000	68545302	6868.4	72.0	0	1384	99.0%
N_004	93800000	67799408	6786.6	73.0	0	1698	99.0%
N_005	127200000	85026502	8525.8	90.0	0	2325	99.0%
N_006	92200000	66514522	6663.8	71.0	0	1323	99.0%
N_007	118400000	82530358	8266.4	89.0	0	2273	98.0%
N_008	103400000	73679224	7374.9	79.0	0	1971	97.0%
N_009	91600000	65722498	6583.9	69.0	0	1350	99.0%
N_010	108600000	75574066	7569.6	79.0	0	1759	99.0%
N_011	113400000	81175626	8133.7	85.0	0	2211	98.0%
N_012	100000000	70092172	7017.5	75.0	0	1770	99.0%
N_013	110400000	75176846	7534.2	80.0	0	1839	99.0%
N_014	112200000	78358826	7854.8	83.0	0	1906	99.0%
N_015	91000000	63545944	6368.9	67.0	0	1685	99.0%
N_016	91800000	65857092	6596.5	70.0	0	1663	99.0%
N_017	87000000	62389988	6250.1	66.0	0	1547	99.0%
N_018	100600000	69545786	6973.1	74.0	0	1634	99.0%
N_019	99600000	70226170	7044.3	75.0	0	1694	99.0%
N_020	97000000	69157374	6938.6	74.0	0	1460	99.0%
N_021	110800000	76755234	7695.4	81.0	0	1818	99.0%
N_022	99600000	69107514	6921.2	73.0	0	1654	99.0%
N_023	96000000	67600640	6779.7	71.0	0	1674	99.0%
N_024	103800000	72976592	7318.2	76.0	0	1618	99.0%
N_025	111800000	94807756	7367.5	77.0	0	2181	99.0%
N_026	94000000	79919110	8025.0	86.0	0	2093	99.0%
N_027	108400000	90379644	8233.4	88.0	0	1975	99.0%

N_028	85200000	72333346	7067.5	75.0	0	1501	99.0%
N_029	100000000	84119900	10069.2	106.0	0	2050	99.0%
N_030	88000000	75626602	7989.7	84.0	0	1923	99.0%
N_031	89800000	76411742	7220.4	77.0	0	1833	99.0%
N_032	85200000	72704462	8214.2	87.0	0	1686	99.0%
N_033	102600000	73471438	9152.0	93.0	0	1627	99.0%
N_034	115400000	80027658	9446.0	96.0	0	1909	99.0%
N_035	121000000	82073610	10143.9	104.0	0	2042	99.0%
N_036	99600000	70473708	9564.3	98.0	0	1709	99.0%
N_037	153400000	100333288	8736.7	89.0	0	2575	99.0%
N_038	114400000	79662948	8390.8	87.0	0	1877	99.0%
N_039	101200000	71968384	7971.6	82.0	0	1769	99.0%
N_040	120400000	81830532	9206.5	94.0	0	1933	99.0%
N_041	134000000	91081266	7602.9	76.0	0	2328	99.0%
N_042	138000000	93996252	7225.9	72.0	0	2520	99.0%
N_043	154600000	100914114	7768.1	77.0	0	2403	99.0%
N_044	140600000	95170456	7855.7	77.0	0	2292	99.0%
N_045	127200000	86975448	8774.5	88.0	0	1988	99.0%
N_046	116400000	83567574	8183.3	81.0	0	2001	99.0%
N_047	111000000	79298270	7533.8	75.0	0	1817	99.0%
N_048	133000000	91621982	7997.0	80.0	0	2377	99.0%
N_049	108800000	91529320	8628.7	85.0	0	2373	99.0%
N_050	101800000	85892198	8441.9	83.0	0	2067	99.0%
N_051	114600000	95458088	9656.8	96.0	0	2454	99.0%
N_052	110800000	93078508	8959.2	89.0	0	2080	99.0%
N_053	88600000	75664626	8927.1	88.0	0	1788	99.0%
N_054	106200000	89523476	9465.8	94.0	0	1928	99.0%
N_055	84400000	72372718	7219.5	71.0	0	1701	99.0%
N_056	94200000	80227240	8538.3	84.0	0	2224	99.0%
N_057	92800000	79688898	9476.0	99.0	0	1736	99.0%
N_058	97600000	82872738	7987.6	84.0	0	2076	99.0%
N_059	112200000	94508806	9035.8	96.0	0	2292	99.0%
N_060	119800000	99865990	7249.2	76.0	0	2490	99.0%
N_061	110600000	91512622	8418.9	89.0	0	2415	99.0%
N_062	85400000	73428638	7573.6	79.0	0	1912	99.0%
N_063	109400000	91479158	7641.4	81.0	0	2402	99.0%
N_064	89200000	76276228	7263.2	77.0	0	1720	99.0%
N_065	104600000	75727686	9174.2	94.0	0	1883	99.0%
N_066	101000000	71991364	8607.1	87.0	0	1891	99.0%
N_067	109000000	77367076	9561.6	98.0	0	1876	99.0%
N_068	111200000	78217332	9325.0	95.0	0	1799	99.0%
N_069	129400000	87364950	7585.8	77.0	0	2196	99.0%
N_070	116200000	81610044	8972.8	90.0	0	1933	99.0%

N_071	105800000	75028444	7250.2	74.0	0	1772	99.0%
N_072	112400000	79687240	8036.1	81.0	0	2062	99.0%
N_073	123200000	85907714	7985.4	75.0	0	2206	99.0%
N_074	119000000	84053078	8306.0	79.0	0	2095	99.0%
N_075	139600000	96152396	9469.9	90.0	0	2456	99.0%
N_076	131600000	89221870	10005.3	96.0	0	2286	99.0%
N_077	127600000	88927116	9174.2	87.0	0	2070	99.0%
N_078	136600000	94340742	7352.3	70.0	0	2410	98.0%
N_079	100800000	71907044	9169.3	85.0	0	1743	99.0%
N_080	124400000	85078162	7642.9	72.0	0	2025	99.0%
N_081	99200000	84385714	8447.7	85.0	0	2027	99.0%
N_082	115800000	96872590	9680.4	99.0	0	2307	99.0%
N_083	114000000	96598268	9676.8	97.0	0	2392	99.0%
N_084	110200000	93180314	9329.3	94.0	0	1979	99.0%
N_085	104400000	88409020	8848.6	88.0	0	2103	99.0%
N_086	85600000	72936470	7305.2	74.0	0	1872	99.0%
N_087	106000000	90173836	9030.6	90.0	0	2232	99.0%
N_088	109800000	92587178	9279.5	94.0	0	2197	99.0%
N_089	104000000	86378168	8638.3	87.0	0	2226	99.0%
N_090	104200000	87480814	8741.4	88.0	0	2209	99.0%
N_091	102600000	85605428	8558.1	86.0	0	2246	99.0%
N_092	113800000	94820612	9475.8	95.0	0	2452	99.0%
N_093	80000000	69006124	6893.6	69.0	0	1676	98.0%
N_094	94400000	79796840	7967.8	80.0	0	2383	99.0%
N_095	94000000	79430004	7933.6	80.0	0	2176	99.0%
N_096	88000000	75044702	7497.4	76.0	0	1744	99.0%

Supplementary table 4 –MMR deficiency detected in a population based Norwegian cervical cancer patient cohort (total n=5)

**Case**	**Age**	**Histological type**	**FIGO-18 stage**	**Last status (months follow-up)**	**MLH-1 SI**	**PMS-2 SI**	**MSH-2 SI**	**MSH-6 SI**
Patient 1	62	NEC	IV	Dead from disease (3)	0	0	4	6
Patient 2	42	NEC	I	Alive (125)	0	0	4	9
Patient 3	48	NEC	III	Dead from disease (14)	0*	0*	6*	6*
Patient 4	57	SCC	IV	Alive (92)	2	0	1	6
Patient 5	48	SCC	I	Alive (174)	2	0	6	6

FIGO: International Federation of Gynecology and Obstetrics. SI: staining index, NEC: neuroendocrine carcinoma, SCC: squamous cell carcinoma, ASC: adenosquamous cell carcinoma.
* TMA score

Supplementary table 5 – Clinicopathological characteristics for patients with neuroendocrine carcinomas according to MMR deficiency.

**Variables (n)**	**MMR-P (n= 7)**	**MMR-D (n= 3)**	**p-value [a]**
Median age (10)			0.500
< 45 years	4 (57)	1 (33)	
≥ 45 years	3 (43)	2 (67)	
FIGO-18 stage (10)			
I-IB1	2 (20)	2 (20)	
IB2-IV	8 (80)	8 (80)	
Max tumour diameter (7)			1.000
≤ 4 cm	2 (40)	1 (50)	
> 4 cm	3 (60)	1 (50)	
Depth of invasion (5)			1.000
Low (≤ 7 mm)	1 (33)	1 (50)	
High (> 7 mm)	2 (67)	1 (50)	
Inflammatory reaction (9)			0.635 [b]
No	3 (50)	2 (67)	
Intermediate	3 (50)	1 (33)	
Strong	0 (0)	0 (0)	
Vascular space invasion (8)			1.000
No	3 (75)	1 (50)	
Yes	1 (25)	1 (50)	

pMMR: proficient MMR, dMMR: deficient MMR. FIGO: Fédération Internationale de Gynécologie et d'Obstétrique. [a] Fisher exact, [b] chi-square.

Supplementary table 6 - Clinicopathological characteristics for patients with MSH-2 high vs low tumors.

**Variables (n)**	**MSH-2 Low (n= 48)** n (%)	**MSH-2 High (n= 410)** n (%)	**p-value** [a]
Median age (458)			0.585
< 45 years	22 (46)	205 (50)	
≥ 45 years	26 (54)	205 (50)	
FIGO-18 stage (458)			0.937
I-IB1	16 (33)	139 (34)	
IB2-IV	32 (67)	271 (66)	
Max tumor diameter in MRI (243)			0.587
≤ 4 cm	12 (55)	134 (61)	
> 4 cm	10 (45)	87 (39)	
Histologic type (458)			0.083
Squamous cell carcinoma	35 (73)	306 (75)	
Adenocarcinoma	7 (15)	79 (19)	
Adenosquamous carcinoma	4 (8)	10 (2)	
Neuroendocrine carcinoma	2 (4)	6 (2)	
Undifferentiated	0 (0)	9 (2)	
Grade (452)			0.876
Grade 1/2	42 (88)	350 (87)	
Grade 3	6 (12)	54 (13)	
BMI (452)			0.648
≤ 25	24 (9.8)	221 (55)	
> 25	23 (11.1)	184 (45)	
Inflammatory reaction (442)			0.425
No	8 (18)	59 (15)	
Intermediate	35 (78)	297 (75)	
Strong	2 (4)	41 (10)	
Vascular space invasion (333)		0.838	
No	28 (68)	204 (70)	
Yes	13 (32)	88 (30)	
P53 status (253)			0.005
Integral	19 (54)	154 (71)	
Negative	9 (26)	17 (8)	
High	7 (20)	47 (21)	

The number of cases in each group is given followed by percentage for each column in parenthesis.
FIGO: Fédération Internationale de Gynécologie et d'Obstétrique. BMI: body mass index
[a] Chi-square

Supplementary table 7 – List of top-20 enriched genesets in MSH-2 low tumors in C5 and 11 enriched genesets in Hallmarks

Gene Set	Size	ES	NES	Non pvalue	FDR (%)
**c5**					
GO_CORNIFICATION	110	0,61	3,19	0	0
GO_RESPONSE_TO_TYPE_I_INTERFERON	92	0,63	3,07	0	0
GO_T_CELL_ACTIVATION_INVOLVED _IN_IMMUNE_RESPONSE	98	0,58	2,97	0	0
GO_MHC_PROTEIN_COMPLEX	21	0,84	2,96	0	0
GO_ADAPTIVE_IMMUNE_RESPONSE	376	0,49	2,95	0	0
GO_GRANULOCYTE_MIGRATION	126	0,55	2,93	0	0
GO_ANTIGEN_BINDING	53	0,64	2,92	0	0
GO_CCR_CHEMOKINE_RECEPTOR_BINDING	37	0,71	2,92	0	0
GO_NEUTROPHIL_MIGRATION	105	0,57	2,92	0	0
GO_CYTOKINE_SECRETION	207	0,51	2,91	0	0
GO_CHEMOKINE_RECEPTOR_BINDING	59	0,62	2,9	0	0
GO_TYPE_2_IMMUNE_RESPONSE	35	0,7	2,9	0	0
GO_REGULATION_OF_CELL_KILLING	88	0,58	2,89	0	0
GO_INTERFERON_GAMMA_MEDIATED_ SIGNALING_PATHWAY	84	0,56	2,82	0	0
GO_RESPONSE_TO_INTERFERON_GAMMA	186	0,5	2,82	0	0
GO_INTERLEUKIN_1_BETA_SECRETION	48	0,64	2,81	0	0
GO_RESPONSE_TO_PROTOZOAN	18	0,82	2,79	0	0
GO_LYMPHOCYTE_CHEMOTAXIS	56	0,61	2,78	0	0
GO_INTERLEUKIN_1_BETA_PRODUCTION	77	0,58	2,78	0	0
GO_POSITIVE_REGULATION_OF_CYTOKINE_SECRETION	126	0,52	2,78	0	0
**Hallmarks**					
HALLMARK_INTERFERON_ALPHA_RESPONSE	92	0,71	3,57	0	0
HALLMARK_INTERFERON_GAMMA_RESPONSE	195	0,63	3,55	0	0
HALLMARK_ALLOGRAFT_REJECTION	199	0,58	3,33	0	0
HALLMARK_INFLAMMATORY_RESPONSE	195	0,54	3,11	0	0
HALLMARK_TNFA_SIGNALING_VIA_NFKB	195	0,51	2,9	0	0
HALLMARK_IL6_JAK_STAT3_SIGNALING	85	0,56	2,76	0	0
HALLMARK_COMPLEMENT	193	0,35	1,95	0	0,04
HALLMARK_COAGULATION	133	0,33	1,81	0	0,11
HALLMARK_KRAS_SIGNALING_UP	190	0,32	1,81	0	0,1
HALLMARK_IL2_STAT5_SIGNALING	194	0,3	1,68	0	0,38
HALLMARK_P53_PATHWAY	194	0,28	1,55	0	0,83
